# Successful use of reduction aortoplasty in patients undergoing aortic valve replacement with ascending aortic dilatation.

**DOI:** 10.1186/1749-8090-10-S1-A270

**Published:** 2015-12-16

**Authors:** Kardo Ale'aldeen, Subir Datta, Ragheb Hasan

**Affiliations:** 1 School of Medicine, University of Manchester, Oxford Road, Manchester, M13 9PL, UK; 2 Department of Cardiac Surgery, Manchester Royal Infirmary, Oxford Road, Manchester, M13 WL, UK

## Background/Introduction

Ascending aortic dilatation is associated with bicuspid aortic valve disease. Some advocate replacing the ascending aorta in conjunction with aortic valve replacement. We adopted to perform ascending aortoplasty at the time of aortic valve replacement.

## Aims/Objectives

The aim of this study was to analyze the short and long term post-operative outcomes in this group of patients.

## Method

We performed a retrospective analysis on all patients who underwent reduction aortoplasty during aortic valve surgery from 2005 to 2013. This was a single surgeon and single center study. Data was collected from the departmental database and office of national statistics. Study end-points included early and late mortality and incidence of aortic dissection.

## Results

We identified 26 patients who underwent aortoplasty during aortic valve replacement. Age (66 ± 13 years), sex (male: female, 15:11), euroscore (5 ± 3), bypass time (95 ± 18 min), xclamp time (68 ± 13 min). Native valve pathology (stenosis: regurgitation: mixed, 13:4:9). Type of implant (mechanical: biological, 13:13). Median prosthetic valve size was 25 mm in all patients. Post-operatively there was no incidence of stroke. Two patients required hemofiltration but recovered on discharge. Early mortality was (n = 1, 4%) and 10 year mortality was (n = 6, 23%). There was no incidence of aortic dissection.

## Discussion/Conclusion

In our study, aortoplasty to counter aortic dilatation was successfully performed with acceptable levels of morbidity and mortality. Reduction of the aortic root during the procedure did not give rise to post-operative aortic dissection.

